# K-Track-Covid: interactive web-based dashboard for analyzing geographical and temporal spread of COVID-19 in South Korea

**DOI:** 10.3389/fpubh.2024.1347862

**Published:** 2024-04-26

**Authors:** Hanbyul Song, Kyulhee Han, Jiwon Park, Zhe Liu, Taewan Goo, Ashok Krishnamurthy, Taesung Park

**Affiliations:** ^1^Interdisciplinary Program in Bioinformatics, Seoul National University, Seoul, Republic of Korea; ^2^Department of Mathematics and Computing, Mount Royal University, Calgary, AB, Canada; ^3^Department of Statistics, Seoul National University, Seoul, Republic of Korea

**Keywords:** COVID-19, South Korea, R shiny, interactive dashboard, mathematical model, infectious disease

## Abstract

The COVID-19 pandemic has necessitated the development of robust tools for tracking and modeling the spread of the virus. We present ‘K-Track-Covid,’ an interactive web-based dashboard developed using the R Shiny framework, to offer users an intuitive dashboard for analyzing the geographical and temporal spread of COVID-19 in South Korea. Our dashboard employs dynamic user interface elements, employs validated epidemiological models, and integrates regional data to offer tailored visual displays. The dashboard allows users to customize their data views by selecting specific time frames, geographic regions, and demographic groups. This customization enables the generation of charts and statistical summaries pertinent to both daily fluctuations and cumulative counts of COVID-19 cases, as well as mortality statistics. Additionally, the dashboard offers a simulation model based on mathematical models, enabling users to make predictions under various parameter settings. The dashboard is designed to assist researchers, policymakers, and the public in understanding the spread and impact of COVID-19, thereby facilitating informed decision-making. All data and resources related to this study are publicly available to ensure transparency and facilitate further research.

## Introduction

Although the COVID-19 pandemic is no longer acute, its effects on health, economy, and society remain significant, highlighting the need to understand various aspects of its recovery and analyze its impacts (1). Governments and institutions worldwide are in need of sophisticated tools to track, understand, and manage the spread of the virus (1). Numerous epidemiological dashboards like those from the World Health Organization (WHO) (2), Johns Hopkins University’s (JHU) Center for Systems Science and Engineering (CSSE) (3), and the Centers for Disease Control and Prevention (CDC) (4, 5) provide valuable insights into global COVID-19 trends.

The WHO dashboard, for instance, offers daily global counts of cases, deaths, and vaccine distribution focusing more on the global scale. Similarly, the JHU CSSE Dashboard stands out for its geospatial representation and data granularity, focused on the United States (3). In addition to cases, deaths, and recoveries data, it has incorporated other metrics like case-fatality ratios and testing data. Although it offers three levels of administrative data granularity—countries, and states/provinces—this fine-grained detail is primarily available only for the United States. Furthermore, while the dashboard provides valuable data, its map representation is limited to the most recent date, without displaying changes over time (3). The CDC’s COVID-NET dashboard offers different topics to see specific data trends. These topics include rates by age group, sex, race, ethnicity, and clinical characteristics. While the data can be displayed in a graph or as a table, a map representation is not provided (4). Lastly, the Africa CDC’s COVID-19 Africa Hotspot Dashboard helps to identify countries that have growing or widespread outbreaks of COVID-19. It is tailored to be highly sensitive in detecting potential hotspots, acknowledging the trade-off in specificity (5).

Existing dashboards often lack forecasting capabilities, essential for policymakers and health professionals to prepare and strategize based on potential future trends. Most current tools fail to offer these predictive insights, highlighting a limitation that needs to be addressed. To address the limitations of current analytical tools, we present ‘K-Track-Covid,’ a web-based interactive dashboard developed with the R Shiny package (6). This dashboard offers insights into the epidemiological situation and features a robust forecasting function. Its predictive power comes from incorporating advanced mathematical models to forecast the pandemic’s trajectory, which we will outline briefly in the Methods section.

The ‘K-Track-Covid’ dashboard focuses on South Korea—a nation that has been at the forefront of proactive and robust public health interventions, such as comprehensive contact tracing and testing (7). This targeted approach allows the dashboard to delve into complexities often glossed over in more broad-scope dashboards (2, 3). Unlike global dashboards that provide periodic global snapshots, ‘K-Track-Covid’ allows for detailed examination of South Korea-specific data across various times, facilitating time- and location-based analyses. This feature supports stakeholders, from researchers to policymakers, in making informed decisions. ‘K-Track-Covid’ represents a significant advancement in digital epidemiological tools, offering a detailed lens on South Korea’s COVID-19 situation.

Furthermore, the ‘K-Track-Covid’ dashboard features a highly modular and scalable architecture, allowing easy customization to various countries or regions. The key to this adaptability is access to robust and reliable local data. With the integration of such data, the dashboard transforms into a versatile instrument for conducting detailed, localized COVID-19 analysis, regardless of location.

The dashboard offers four main functions: (1) it automatically updates COVID-19 data in South Korea, including case counts (confirmed and deceased), according to the available data released by the government. (2) It allows for daily and cumulative tracking, helping to project short and long-term trends. This feature is designed to resume full functionality should the government reinitiate the release of COVID-19 data. (3) The dashboard presents data at various geographical resolutions, enhanced with socio-demographic metrics such as medical resources and population profiles, for detailed spatial analyses. (4) Most notably, it integrates a mathematical model to forecast and visually illustrate the disease’s spread over time on a dynamic MP4 animated map. This unique feature allows users to anticipate hotspots and track regional progression, providing an engaging experience. This combination of features makes it a comprehensive tool for academics, policymakers, and the public interested in South Korea’s pandemic trajectory.

In a time of abundant yet complex data, ‘K-Track-Covid’ aims to be a reliable, user-friendly resource for all audiences. The remainder of this paper is structured to offer a detailed account of ‘K-Track-Covid.’ Following this Introduction, the Methods section will elucidate the technical architecture, data sources, and the overall structure of the dashboard. The Results section will focus on the dashboard’s functionalities, user engagement, and impact, including a comparison with other available dashboards to contextualize its utility and performance. In the Discussion, we will explore broader implications for pandemic management in South Korea, as well as the adaptability of such a tool for other regions or future public health crises.

## Materials and methods

### Data sourcing

#### Regional data

Data Sourcing is an essential part of our dashboard, ensuring accurate COVID-19 information for South Korea. We use an Application Programming Interface (API) from the South Korean government’s data portal, provided by the Korea Disease Control and Prevention Agency (KDCA) (8). To manage this data, we utilize R libraries, employing ‘httr’ (9) for Hyper Text Transfer Protocol (HTTP) requests and ‘xml2’ (10) for parsing Extensible Markup Language (XML) data. This combination ensures consistent and standardized data that is ready for analysis.

Our dataset covers the period from the pandemic’s start in early 2020 to September 2023, offering a broad temporal range for analysis. Initially, data updates occurred daily until May 2023, transitioning to weekly updates from that point forward. On September 1, 2023, the Korean government reclassified COVID-19 as a Class 4 infectious disease, leading to the discontinuation of regular updates to the provincial incidence data (8). We primarily use the daily data to ensure detailed analyses. This dataset spans all 17 of South Korea’s major administrative regions, like Seoul, Busan, and Daegu, covering metrics such as confirmed cases, deaths, and outbreak status per area. Such granularity provides a precise regional perspective on the pandemic, which is crucial for academic study and policymaking.

In addition to the COVID-19 data, we also incorporate regional socio-demographic data obtained from the Korean Statistical Information Service (KOSIS) (11). These data provide demographic insights such as age distribution, gender, and smoking rate, which are crucial for a nuanced understanding of the pandemic’s impact. Including KOSIS data enables a more comprehensive analysis, as socio-demographic factors are significant determinants of infection rates and health outcomes. This multi-dimensional approach not only informs targeted public health strategies but also enriches academic discourse on the effects of COVID-19 across diverse population strata within South Korea.

#### Spatial demographic data

Our dashboard enhances epidemiological analysis with spatial demographic data from WorldPop (12). This source offers detailed population density maps at 1 km × 1 km and 100 m x 100 m resolutions globally, useful for addressing census gaps and supporting UN agencies’ planning efforts. In ‘K-Track-Covid,’ this data helps analyze COVID-19 spread in relation to population densities and demographics, especially in densely populated areas like Seoul, Busan, and Daegu. Integrating these maps provides richer, more contextually informed insights, aiding in developing effective public health strategies.

### Architecture

The ‘K-Track-Covid’ dashboard is developed using the RStudio Shiny framework for its reliability, user-friendliness, and advanced data handling capabilities. We employ key R packages for efficient operations: dplyr (13) and tidyverse (14) for data management, sf (15) and raster (16) for geospatial data, plotly (17) for interactive, and ggplot2 (18) for static visualizations. Geospatial interactivity is enhanced with leaflet (19) and leaflet. Extras (20). The architecture combines Shiny with shinydashboard (21) for web interactivity, dividing into a User Interface (UI) and a server-side function. While primarily R-based, we incorporate JavaScript, Cascading Style Sheets (CSS), and Hyper Text Markup Language (HTML) to augment functionality and design.

### Dashboard structure

The ‘K-Track-Covid’ dashboard is structured based on three main tabs: Map, Regional Trend, and Simulation. Table 1 contains the description of each main tab.

**Table 1:** K-Track-Covid dashboard structure.

Main tab	Description
Map	The Map tab incorporates leaflet to display geographical information. A leaflet map is presented on this tab, which covers the entire window size.
Regional Trend	Focuses on visual analytics at a more granular level. It utilizes the plotly package to render the plots.
Simulation	Integrates mathematical models to simulate COVID-19 scenarios based on user-defined parameters.

The dashboard’s user interface is crafted for easy navigation, featuring dropdown menus for region selection, timeframe buttons, and interactive graphs and maps. Tooltips offer extra details when hovering over data points.

In the development phase of the ‘K-Track-Covid’ dashboard, we integrated a user feedback mechanism to refine and enhance the platform’s usability and functionality continually. This feedback mechanism, in the form of a user rating form, is modeled on the Mobile App Rating Scale (21), a validated tool for assessing the quality of health mobile apps. Our adaptation of this scale allows users to rate their experience across multiple dimensions: ease of use, reliability, quality, scope of information, and aesthetics. This approach enables us to gather actionable insights into the dashboard’s performance from the user’s perspective, guiding future improvements.

#### Map tab

**Figure 1:**
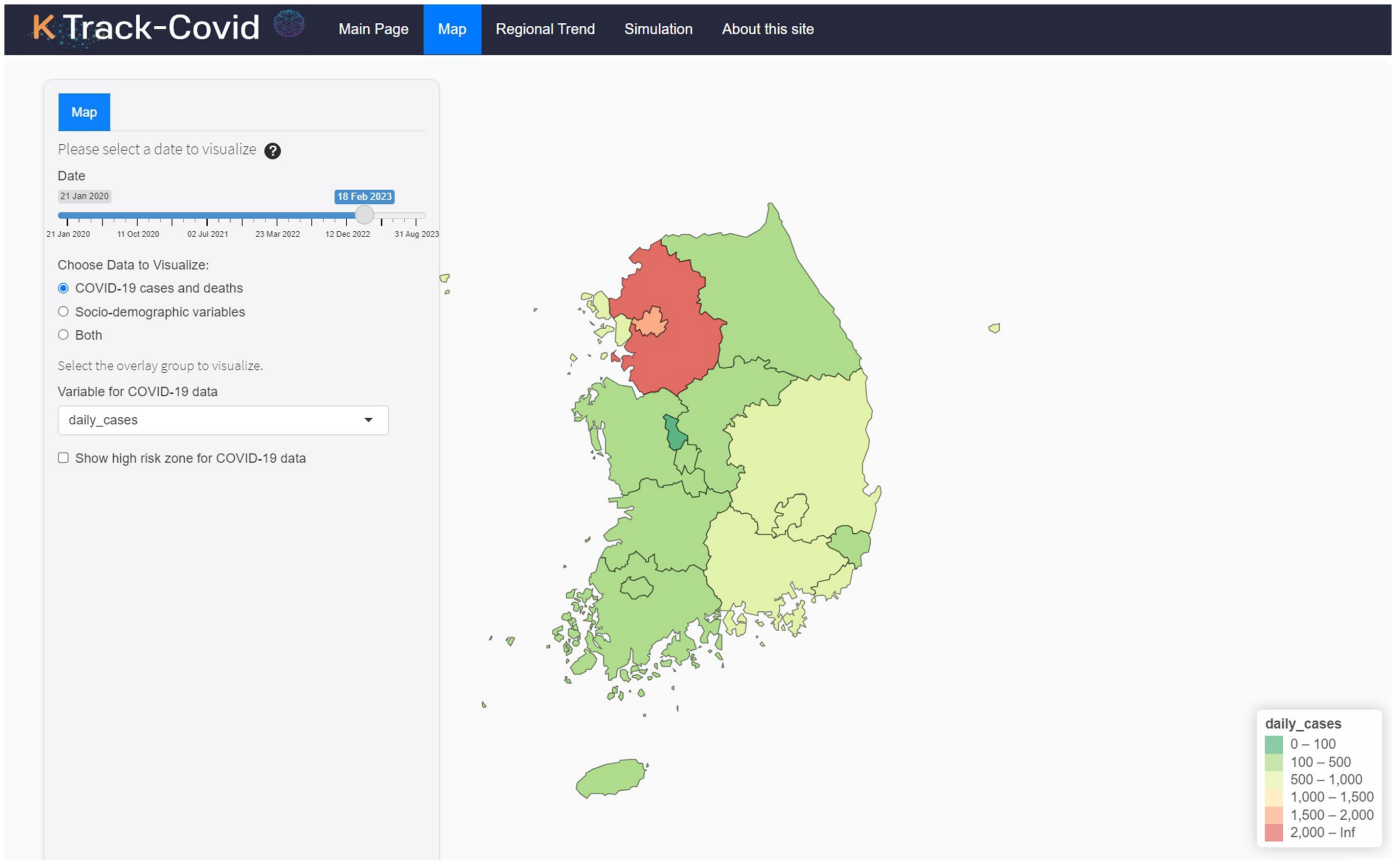
Overview of the K-Track-Covid dashboard’s Map tab.

The Map tab (Figure 1) in ‘K-Track-Covid’ employs Leaflet technology to create an interactive geospatial display. Situated on the left-hand side, a fixed yet draggable absolute panel hosts various control elements, such as a date range slider, a dropdown for overlay options, and selection boxes for additional output. The date range slider allows users to navigate different time periods, providing a temporal dimension often missing from other dashboards. When a user positions their cursor over a particular region on the map, the system displays detailed data about the region. This information can include metrics like daily cases and deaths, each available in raw counts, proportions, and per 100 k population formats.

One of the standout features of the Map tab is its capability to show the spread of the disease with the regional socio-demographic data, including the total number of beds, doctors, seniors, and foreigners per 1,000 people, alongside smoking rates and the total population segmented by age group and gender. Integrating these metrics allows for identifying hotspots and high-risk zones by setting thresholds on metrics like senior population percentages, smoking rates, and healthcare availability. Users can select these metrics to visualize risk levels across regions, enhancing resource allocation and intervention strategies. This feature complements existing COVID-19 data, offering a fuller risk assessment tool for public health decision-making.

The leaflet R package supports the dashboard’s geospatial visualizations, utilizing OpenStreetMap (22) for base maps. The interface has been enhanced with custom CSS styling for intuitive navigation and aesthetic appeal, improving the control panel’s functionality and user interaction with the map.

#### Regional trend tab

**Figure 2:**
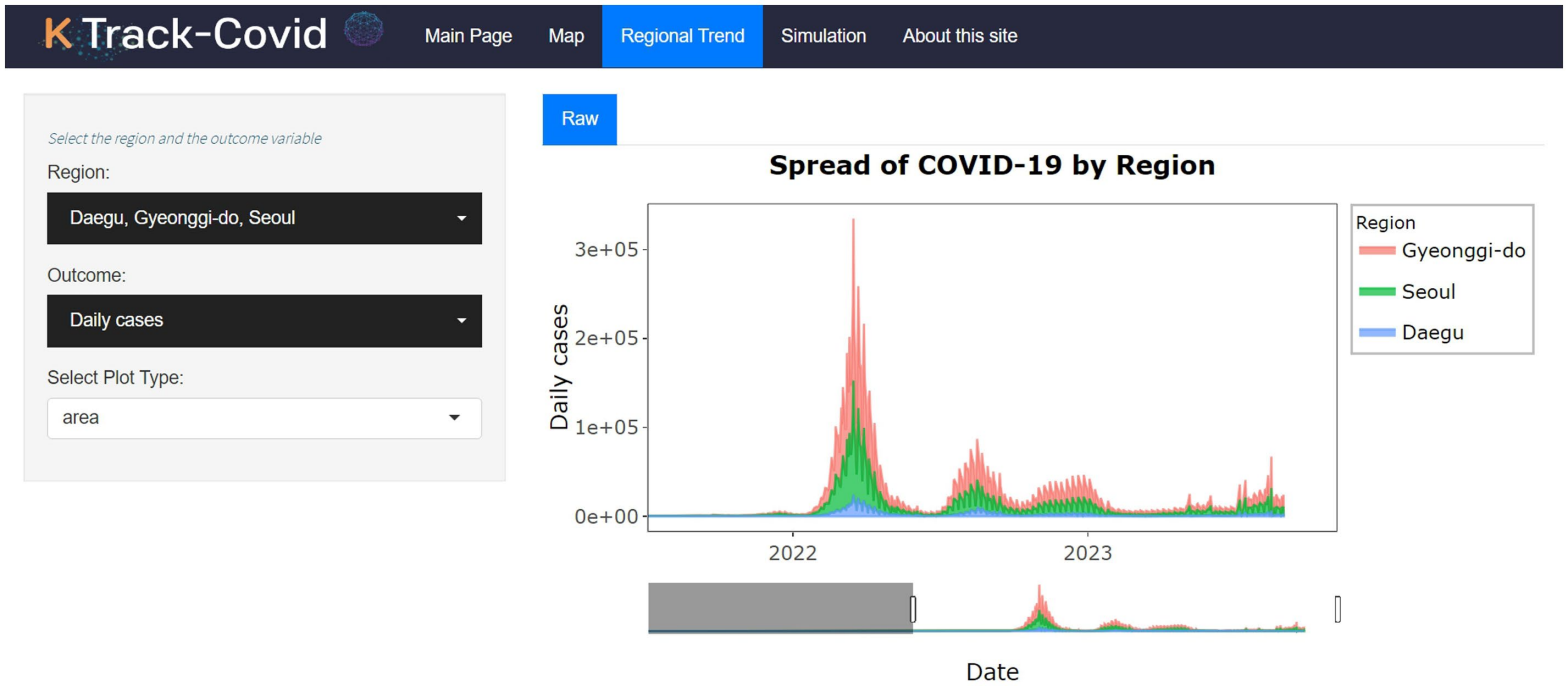
User interface and features of the Regional Trend tab.

The Regional Trend tab (Figure 2) offers users a diverse range of time-series visualization options rendered using the plotly package (17). It provides a dynamic look at the temporal trends of COVID-19 metrics for individual regions within South Korea. Unlike the Map tab, which shows a single moment, the Regional Trend tab provides a comprehensive historical perspective, highlighting changes and trends in the pandemic’s progression across different regions. This approach helps identify patterns, seasonal variations, and outliers in data such as new cases, new deaths, and cumulative figures. The underlying data for these visualizations, drawn from KDCA, has been pre-processed to reflect accurate, region-specific statistics.

The sidebar panel includes a region and an outcome selector dropdown, acting as auxiliary tools to refine the time-series plot according to user preferences. This feature allows users to concentrate on particular regions and outcomes, adding depth and offering a detailed view essential for scientific research and policy development. Consequently, the Regional Trend tab provides a deeper, time-sensitive insight into the pandemic’s effects across the varied landscapes of South Korea.

#### Simulation tab

**Figure 3:**
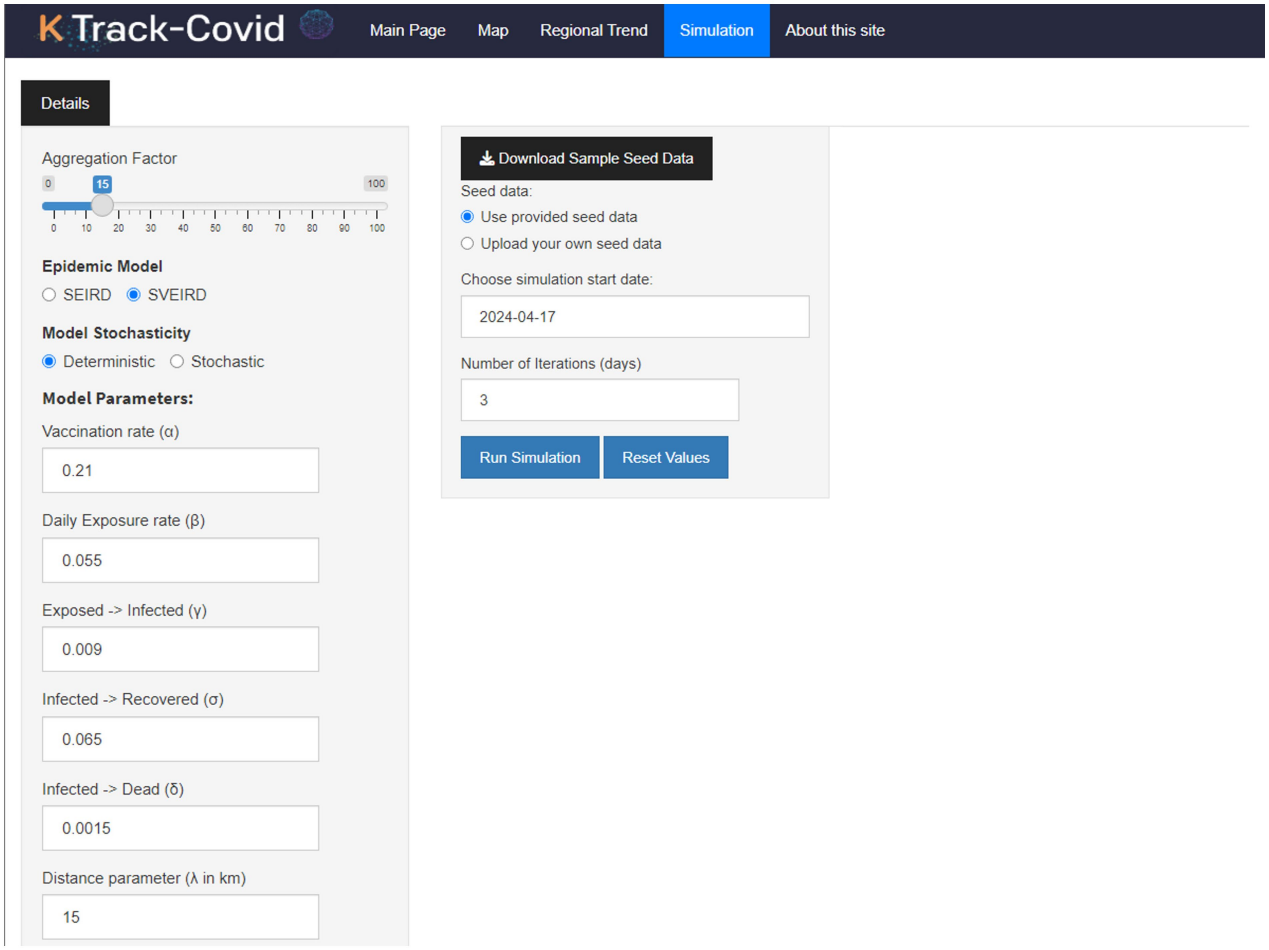
Initial overview of the Simulation tab, displaying the control panel and default outputs.

The Simulation tab (Figure 3) stands out as a pivotal aspect of our dashboard, delivering simulations for projecting COVID-19 scenarios through shinyJS (23) for enhanced UI functionality. It includes a customization sidebar, allowing users to choose between the Susceptible-Exposed-Infectious-Recovered-Deceased (SEIRD) (24, 25) and Susceptible-Vaccinated-Exposed-Infectious-Recovered-Deceased (SVEIRD) (26, 27) models for forecasting. This capability lets users explore future pandemic trends and outcomes based on user-defined parameters, providing a dynamic, personalized simulation experience. This unique functionality, not found in similar tools, significantly enhances our dashboard’s analytical depth, offering a nuanced approach to disease spread prediction.

The SEIRD model enriches the traditional Susceptible-Infectious-Recovered (SIR) framework by integrating ‘exposed’ and ‘deceased’ compartments, allowing for a more detailed understanding of disease progression (28). Expanding upon the SEIRD framework, the SVEIRD model introduces a ‘vaccinated’ compartment to capture the dynamics of vaccination within the population (26). This addition allows for the simulation of vaccination outcomes alongside natural disease progression, providing a comprehensive view of potential interventions. A crucial aspect of both models is incorporating a weight function that calculates the impact of infectious individuals on susceptible individuals based on spatial distribution (29).

The dashboard also includes a feature for stochastic simulation, which introduces probabilistic variations to the model outcomes. This is facilitated by adjustable parameters such as transmission rate (β), recovery rate (σ), and the rate at which exposed individuals become infectious (γ). Users have the flexibility to customize their simulations further by uploading their seed data or utilizing pre-loaded datasets.

In the output section of the Simulation tab, users are provided with textual and tabular summaries that detail the selected epidemiological model’s characteristics. This section aims to offer a clear and structured understanding of the model’s implications without delving into the mathematical equations, focusing instead on the outcomes and interpretations crucial for public health planning and response strategies.

## Results

The multi-dimensional analysis capabilities incorporated into our COVID-19 dashboard serve to elucidate various facets of the pandemic within South Korea. Our dashboard integrates epidemiological, temporal, and geographical data, offering insights that enhance our understanding of the virus’s spread and impact. This innovation is at the core of our project, and it significantly advances current methodologies for COVID-19 analytics. As highlighted in the Materials and Methods section, please be advised that since the Korean government’s cessation of regular updates of regional incidence data as of September 1, 2023, the Map tab and the Regional Trend tab report the results up to this date.

The ‘K-Track-Covid’ dashboard can be accessed online (https://k-track-covid.shinyapps.io/k-track-covid/, accessed on 12 April 2024). As demonstrated in Figure 1, the dashboard is designed to offer an intuitive and interactive user experience, divided into distinct modules accessible via individual tabs.

### Map tab

**Figure 4:**
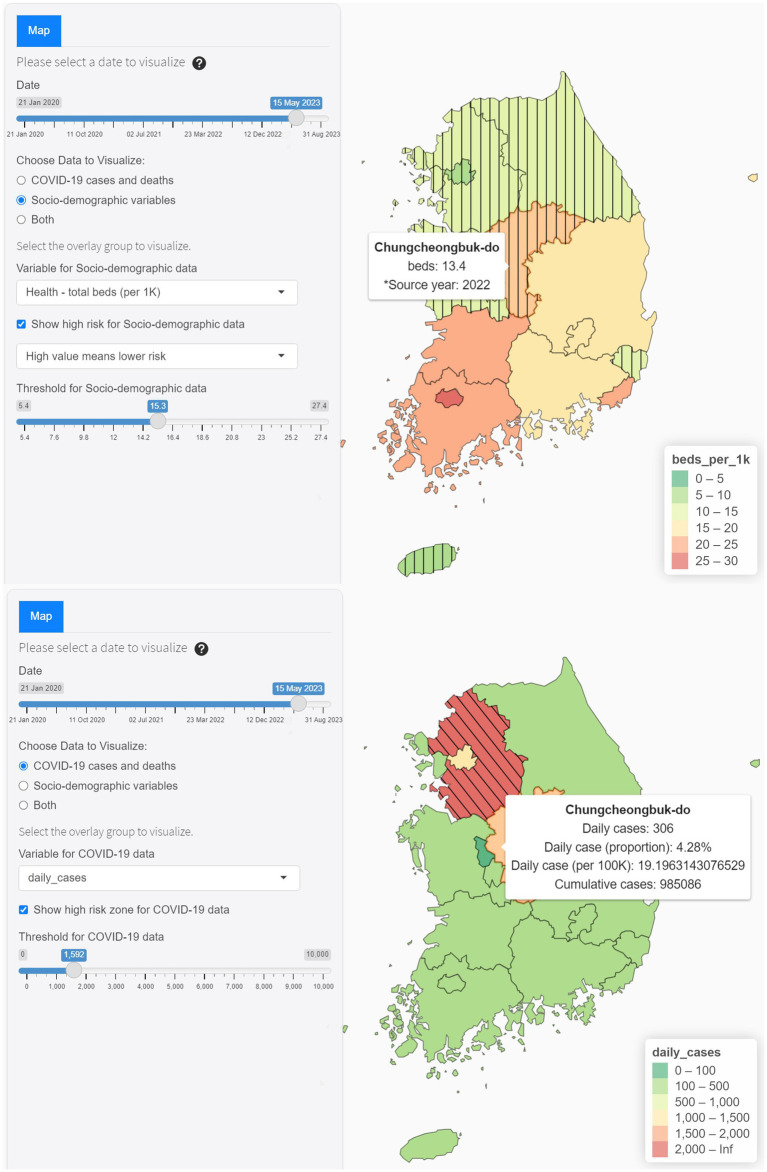
Interactive features of Map tab.

As shown in Figure 4, the Map tab offers geospatial analytics across multiple levels, allowing users to explore daily case numbers or proportions and available hospital beds at the district level. It highlights at-risk populations, enhancing localized response strategies. The map facilitates temporal analysis through selectable date ranges and enables comparisons of current statistics against historical trends with data overlay. This integration provides a comprehensive view of tracking pandemic dynamics and healthcare capacity.

### Regional trend tab

The Regional Trend tab, illustrated in Figure 2, focuses on more localized data, presenting time-series data visualization at the regional level. Users can customize the visualizations through drop-down menus, choosing from different COVID-19 metrics like daily new cases, cumulative cases, and deaths. The analytics in this tab facilitate comparisons within regions, providing a clearer perspective on the pandemic’s localized impact as it unfolds over time.

### Simulation tab

The Simulation tab, illustrated in Figure 3, describes the dashboard’s advanced features. It uses mathematical models to predict COVID-19. The tab allows for significant customization, including the ability for users to adjust parameters to meet specific analytical needs. A user manual is available to assist new users.

#### Input summary panel

**Figure 5:**
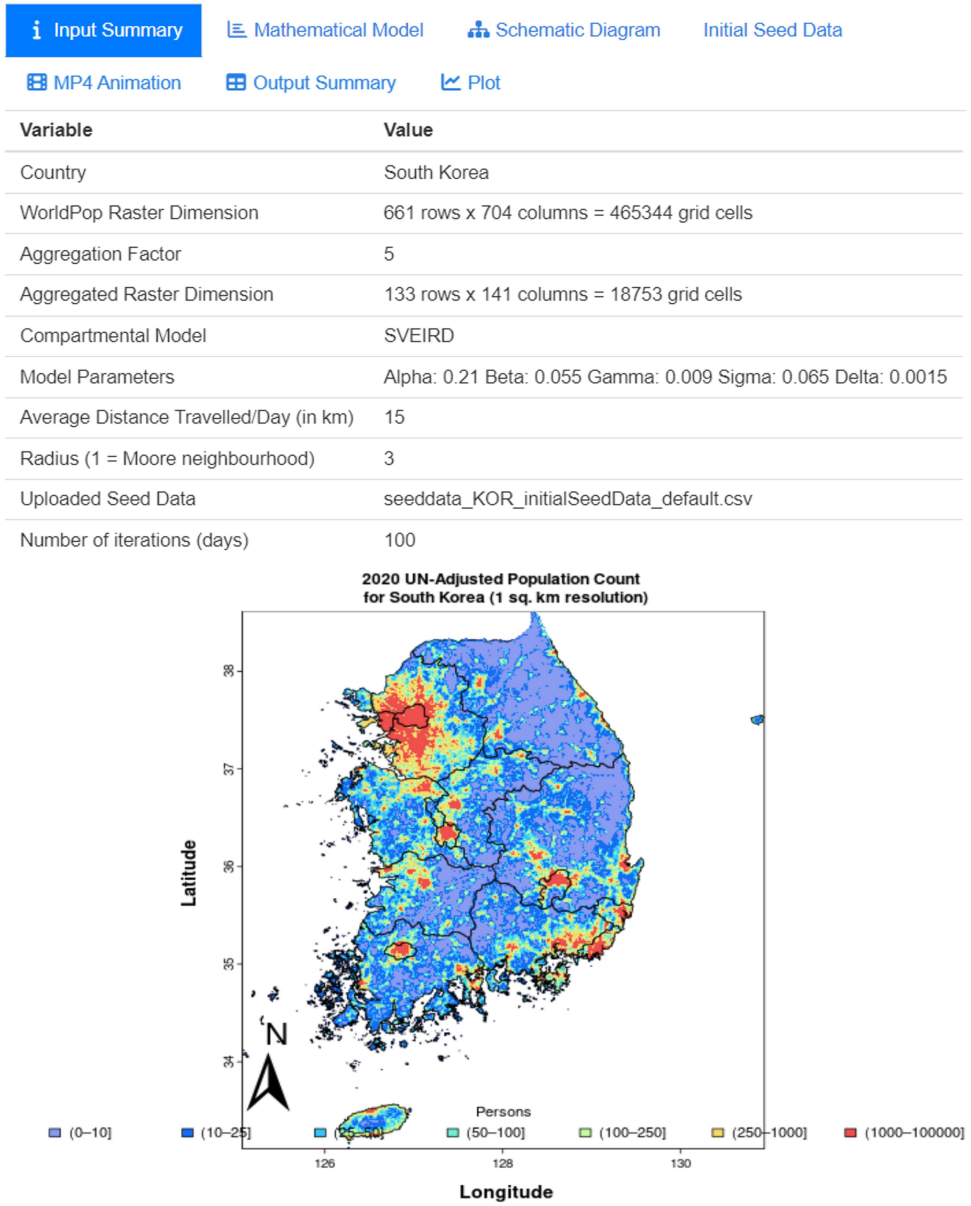
Input summary panel showing user-defined parameters.

Figure 5 provides a snapshot of the Input Summary panel. This panel delivers a comprehensive summary of all user-defined parameters. It serves as a legend and a quick reference, allowing users to verify their configurations before interpreting the simulations.

#### Schematic diagram panel

The Schematic Diagram panel in Supplementary Figure S1 shows a graphical representation of the model’s structure. This diagram outlines the compartmental relationships and flows among Susceptible, Exposed, Infected, Recovered, and Deceased compartments, enhancing understanding of the model’s complexity.

#### Initial seed data panel

**Figure 6:**
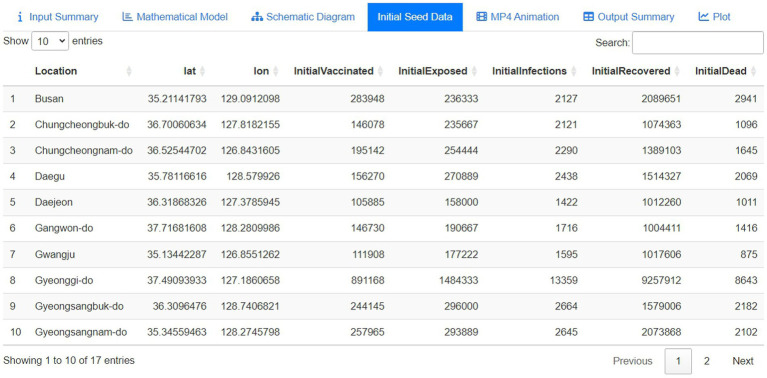
Initial seed data represented by the first 10 rows.

Figure 6 illustrates the initial seed data panel, which visually represents the model input. The seed data encompasses detailed location information, including the region and its corresponding latitude and longitude, followed by initial compartmental values such as vaccinated, exposed, infected, recovered, and deceased individuals. Users have the flexibility to either use the provided seed data or upload their own adjusted seed data. By uploading their own seed data, users can customize the initial conditions for each compartment, tailoring the model to specific regional dynamics.

#### MP4 animation panel

**Figure 7:**
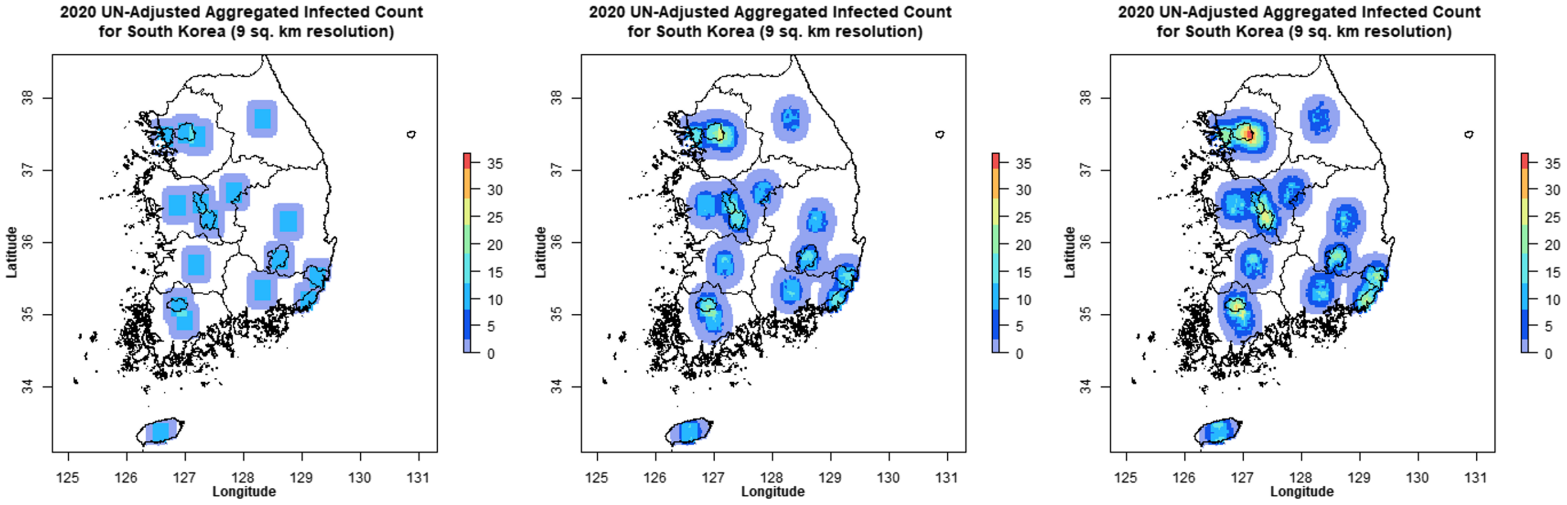
MP4 animation showing the spread of the disease over time.

In Figure 7, the dashboard features an MP4 animation panel that visually demonstrates the epidemiological states’ progression from day one to day 100, covering the entire forecast period. This animation offers a dynamic representation of the model’s predictions over time, making the data more accessible and understandable. Additionally, the animation can be used to forecast and visually illustrate the disease’s spread across the timeframe. For convenience, users can download the animation by clicking on the vertical ellipsis in the right corner of the video.

#### Output summary panel

The Output Summary panel in Supplementary Figure S2 is designed to present a comprehensive tabulation of the model’s predictions, generated based on user-defined inputs and parameters. This is particularly useful for expert users or researchers requiring precise data points for further analysis or reporting. In addition, having a tabular format enables easy data export into other statistical software for subsequent analyses.

#### Plot panel

The Plot panel (Supplementary Figure S3) within the Simulation tab serves as a visual analytics environment, offering a dynamic and interpretable representation of the model’s output metrics over time. Users can download the figure by clicking the ‘Save Image’ button.

### Comparison to existing dashboards

Following the detailed exploration of the ‘K-Track-Covid’ dashboard’s features and capabilities, a comparative analysis is conducted to place its functionalities in context with four other leading COVID-19 dashboards. This comparison is summarized in a comprehensive table (Table 2) that delineates the available functions across all platforms, offering a clear side-by-side view of each dashboard’s unique features and shared capabilities. ‘K-Track-Covid’ provides a comprehensive toolset for regional and socio-demographic analysis with the unique addition of simulation capabilities. However, it underperforms in the global analysis compared to the WHO, JHU, and CDC dashboards. Its commitment to data accessibility and open-source development is on par with the leading dashboards, making it a strong contender in existing COVID-19 dashboards.

**Table 2:** Comparison with publicly available COVID-19 dashboards.

Dashboard	Map representation	Regional analysis	Global analysis	Socio-demographic analysis	Simulation analysis	Data accessibility	Open-source replicability
WHO dashboard	O	X	O	O	X	O	O
JHU dashboard	O	O	O	X	X	O	O
COVID-NET dashboard	X	O	X	O	X	O	X
COVID-19 Africa dashboard	O	X	O	X	X	X	X
K-Track-Covid	O	O	X	O	O	O	O

## Discussion

Our study demonstrates the effectiveness of the ‘K-Track-Covid’ in analyzing COVID-19’s trajectory and impact in South Korea. Its interactive design and analytical capabilities make it useful for researchers, policymakers, and the public. Integrating regional, epidemiological, and spatial demographic data, the dashboard offers a complete view of the pandemic’s spread and effects in South Korea. This localized approach enables an in-depth understanding of COVID-19 dynamics within the country’s specific public health and societal context.

Global platforms like WHO and JHU provide valuable pandemic data on a global scale, while our ‘K-Track-COVID’ dashboard offers in-depth, temporal analysis specific to South Korea. Additionally, our dashboard enhances the socio-demographic analysis, similar to the COVID-NET approach, by displaying it on an interactive map, thus improving the identification of potential high-risk zones. Unlike other platforms, it integrates simulation models for forecasting, which is essential for proactive public health interventions. It also features hotspot identification similar to the COVID-19 Africa dashboard, with adjustable thresholds for cases or deaths to pinpoint emerging risk areas effectively.

An essential feature of ‘K-Track-Covid’ is its integration of socio-demographic data, enabling the identification of potential hotspots and high-risk zones. By incorporating metrics such as the regional distribution of healthcare resources and age demographics, users can pinpoint areas that may face medical constraints or have higher vulnerability. Utilizing these socio-demographic indicators enhances analytical depth and supports targeted interventions for regions needing immediate resources. Another feature of the dashboard is its applicability for future pandemic prevention, preparedness, and response. The Simulation tab can be adjusted to model various infectious diseases by modifying its parameters. For example, the dashboard could be recalibrated to track and analyze the spread of influenza. This flexibility allows health authorities to adapt our models for different infectious diseases rapidly. Furthermore, the tab uses spatial demographic data from WorldPop, which offers population density information at a 1 km by 1 km resolution, enabling an analysis of disease spread relative to population count.

Despite its contributions, we acknowledge the ‘K-Track-Covid’ dashboard’s limitations. Our current version of ‘K-Track-Covid’ is limited to COVID-19 in South Korea. However, it possesses the potential for broader applications. The dashboard can be adapted to model various infectious diseases by incorporating additional geographical and regional data. This adaptability is illustrated through the Map and Regional Trend tabs, which can be recalibrated for other diseases with the necessary data. Furthermore, with access to international geographical information, ‘K-Track-Covid’ could offer insights on a global scale, enhancing infectious disease management worldwide.

In the future, expanding the ‘K-Track-Covid’ dashboard to include other infectious diseases and broadening its scope to various countries is a promising avenue for research. This extension will enable a comprehensive analysis of disease spread and containment strategies across diverse geographic and demographic contexts. Moreover, by integrating real patient data with the enactment of regional policies, mainly through metrics like the Korea Stringency Index (KSI) (30), the dashboard can provide detailed insights into the effectiveness of various public health strategies. Such analysis is pivotal for refining pandemic preparedness and response, enabling the development of targeted, evidence-based policies. Future research will focus on leveraging the KSI to evaluate the relationship between policy stringency and infection trends, aiming to optimize public health interventions for better disease management and prevention.

In conclusion, ‘K-Track-Covid’ is an innovative tool in epidemiological analysis, offering comprehensive insights into the spread and impact of COVID-19 in South Korea. Its predictive capabilities and user-friendly interface make it an asset for a wide range of stakeholders. By facilitating a deeper understanding of the pandemic’s dynamics, ‘K-Track-Covid’ contributes significantly to informed decision-making in public health. As the global community continues to navigate the challenges of the COVID-19 pandemic and future public health crises, tools like ‘K-Track-Covid’ will be essential in guiding effective and timely responses.

## Data availability statement

The original contributions presented in the study are included in the article/Supplementary material, further inquiries can be directed to the corresponding author.

## Author contributions

HS: Writing – original draft, Writing – review & editing, Data curation, Formal analysis, Software, Visualization. KH: Formal analysis, Visualization, Writing – review & editing. JP: Writing – review & editing. ZL: Writing – review & editing. TG: Writing – review & editing, Writing – original draft. AK: Writing – review & editing, Methodology. TP: Writing – review & editing, Conceptualization, Funding acquisition, Methodology, Project administration, Supervision, Writing – original draft.
