# Supplementary material for: K-Track-Covid: interactive web-based dashboard for analyzing geographical and temporal spread of COVID-19 in South Korea
Source: Front Public Health. 2024 Apr 26;12:1347862. doi: 10.3389/fpubh.2024.1347862 (PMC11082270; doi:10.3389/fpubh.2024.1347862)
Supplement: Supplementary file 1 [file Data_Sheet_1.docx]

Supplementary Material

# Supplementary Figures


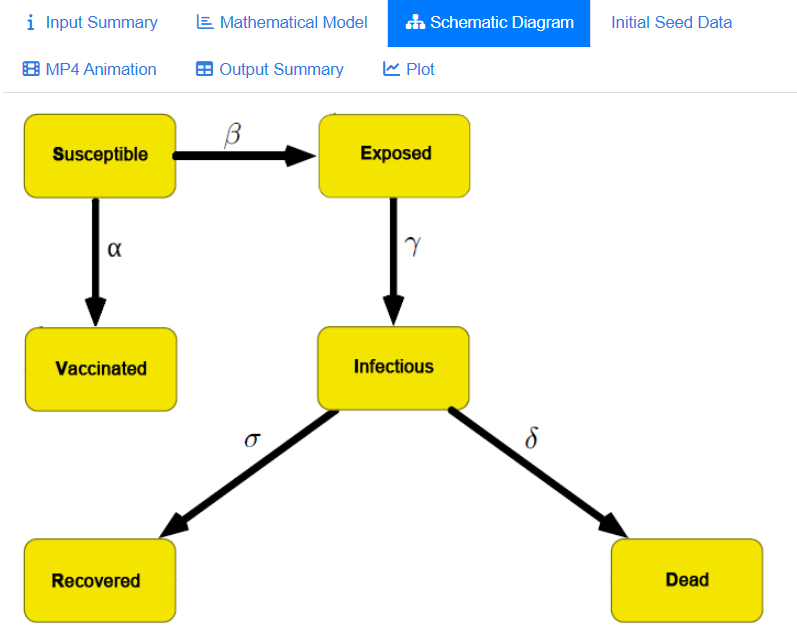


**Supplementary Figure 1.** The Schematic Diagram visually represents the epidemiological model's structure


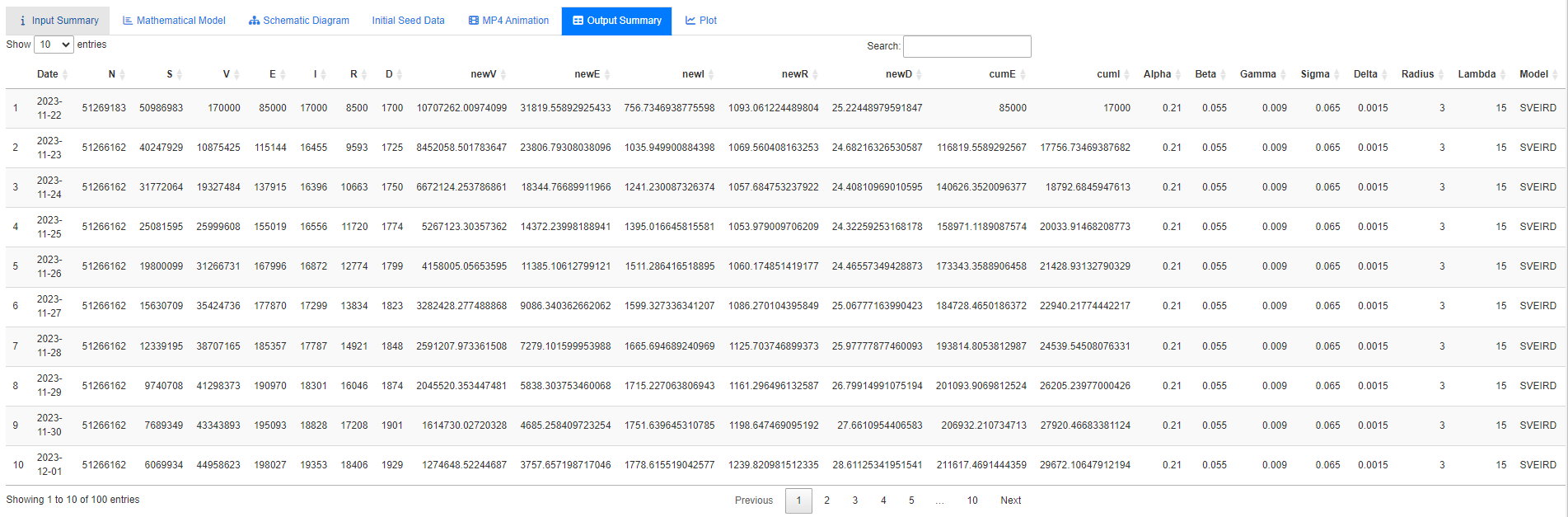


**Supplementary Figure 2.** Structure of the Output Summary Table


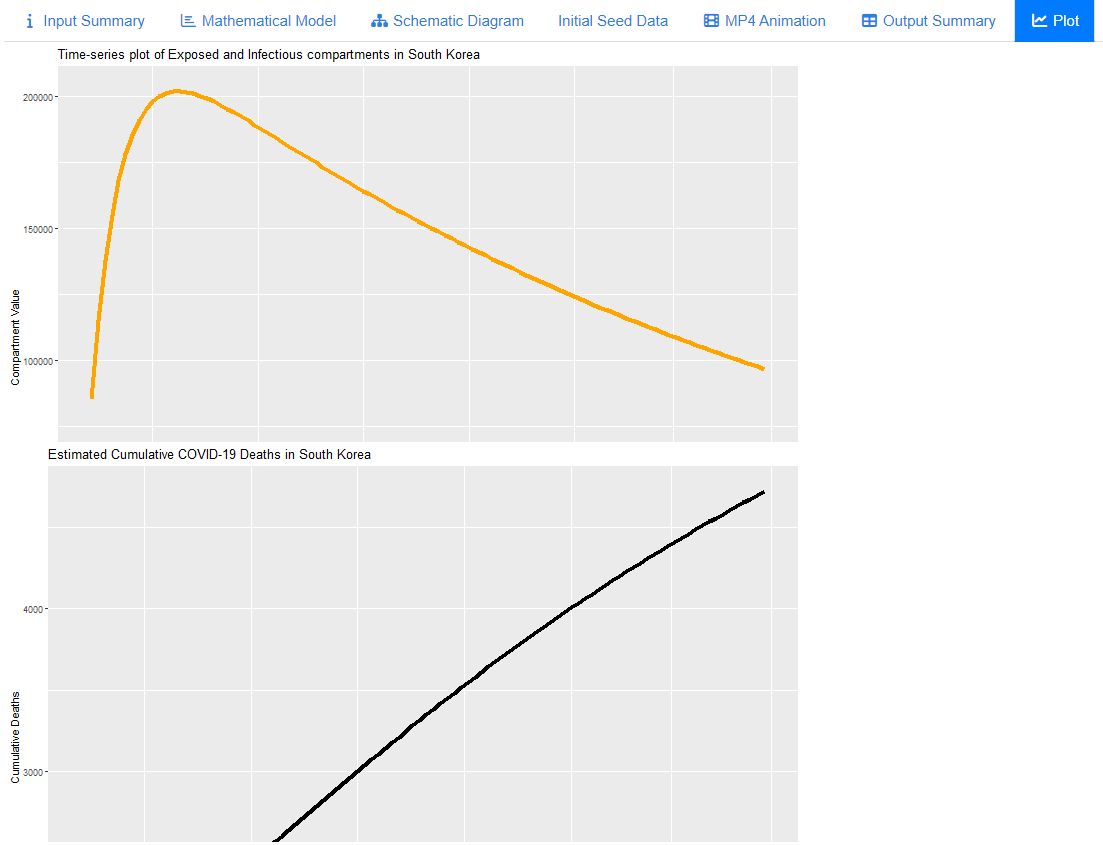

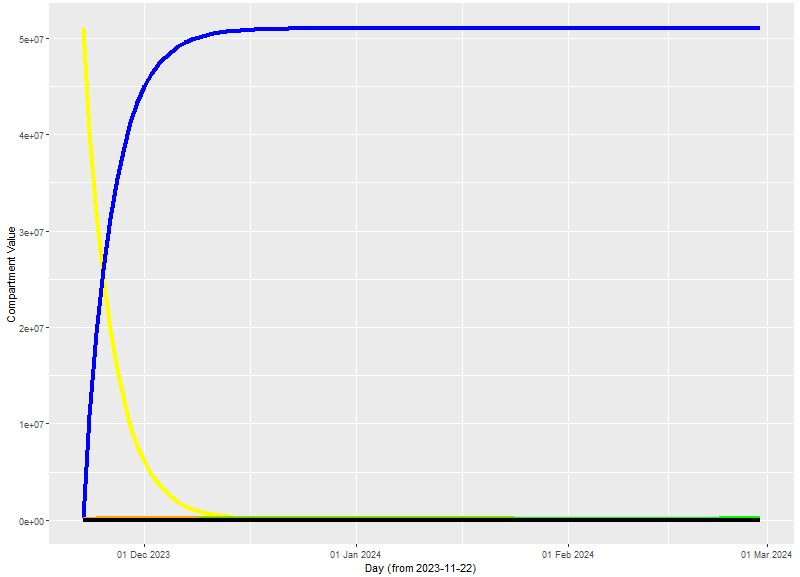


**Supplementary Figure 3.** Visual representation of the Plot panel
